# A Fast Segmentation Method for Fire Forest Images Based on Multiscale Transform and PCA

**DOI:** 10.3390/s20226429

**Published:** 2020-11-10

**Authors:** Lotfi Tlig, Moez Bouchouicha, Mohamed Tlig, Mounir Sayadi, Eric Moreau

**Affiliations:** 1 Member of SIME Laboratory, ENSIT University of Tunis, Tunis 1008, Tunisia; mohamed.tlig@ensit.u-tunis.tn (M.T.); mounirsayadi@yahoo.fr (M.S.); 2 Aix Marseille Univ, Université de Toulon, CNRS, LIS, 83041 Toulon, France; moez.bouchouicha@univ-tln.fr (M.B.); eric.moreau@univ-tln.fr (E.M.)

**Keywords:** Gabor filtering, PCA morphological transformations, fuzzy clustering, color image segmentation, fire forest

## Abstract

Forests provide various important things to human life. Fire is one of the main disasters in the world. Nowadays, the forest fire incidences endanger the ecosystem and destroy the native flora and fauna. This affects individual life, community and wildlife. Thus, it is essential to monitor and protect the forests and their assets. Nowadays, image processing outputs a lot of required information and measures for the implementation of advanced forest fire-fighting strategies. This work addresses a new color image segmentation method based on principal component analysis (PCA) and Gabor filter responses. Our method introduces a new superpixels extraction strategy that takes full account of two objectives: regional consistency and robustness to added noises. The novel approach is tested on various color images. Extensive experiments show that our method obviously outperforms existing segmentation variants on real and synthetic images of fire forest scenes, and also achieves outstanding performance on other popular benchmarked images (e.g., BSDS, MRSC). The merits of our proposed approach are that it is not sensitive to added noises and that the segmentation performance is higher with images of nonhomogeneous regions.

## 1. Introduction

The conventional detection systems of smoke and fire use sensors [1]. One of the major drawbacks, is that the systems do not issue the alarm unless the particles reach the sensors [2]. Recently, as an appropriate alternative to conventional techniques, vision-based fire and smoke detection methods have been adopted. Here, smoke and fire are regarded as a specific kind of texture. It is difficult to accurately detect the appearance of mentioned regions from images due to large variations of color intensities and texture. Although, many research works confirmed that texture features play a very important role in smoke and fire detection [3,4]. A wide recent work demonstrated that the multi-scale based techniques play an important role in smoke and texture classification [5,6]. Developed methods cover both areas; images and videos processing [4,7,8]. In this work, we aim to segment images into significant regions. This will be used to generate useful information for our project. In this paper, we propose a new segmentation approach based on Gabor filtering and Principal Component Analysis (PCA). The proposed method is based on the modification of the superpixels extraction methodology to increase the robustness to added noises and to improve the segmentation accuracy of the fire forests color images. For the extracted features clustering, we used the new version of the fuzzy classifier recently proposed in [9]. Our choice is done regarding the higher performance of the mentioned fuzzy method compared to a large variety of clustering methods; FCM, FGFCM, HMRF-FCM, FLICM, NWFCM, KWFLICM, NDFCM, FR- FCM, and Liu’s algorithm. In [9], Lei et al. introduced a fast fuzzy clustering algorithm to address the problem of computational segmentation complexity of color image segmentation with higher resolution. This was conducted by the use of adaptive local spatial information provided by a pre-segmentation task. Despite its higher accuracy compared to a large number of used algorithms, this method remains limited due to many drawbacks. This is experimentally noted with blurred images and images with nonhomogeneous regions. In this work, our research is focused on the part where we have cited a lower efficiency. It is the task of superpixels extraction. As mentioned above, we have introduced the multiscale image transformation based on the Gabor filtering.

Many applications approved that Gabor feature sets are high-dimensional (typically defined by the number of orientations and frequencies). Concatenating all the feature images tends to exacerbate the dimensionality problems and computing complexity [4,6]. To overcome this issue, we introduce a dimensionality reducer before the superpixels extraction stage. In literature, there are many proposed dimensionality reduction methods; Independent Component Analysis (ICA), Principal Component Analysis (PCA), and Canonical Correlation Analysis (CCA) [10,11]. In this work, we find that PCA is sufficient.

Note that our intention was not to develop an end to end new segmentation approach of color image. Rather, we propose improving this task in general, by integrating several methodologies. As a first goal, these methodologies (multi-resolution filtering, superpixels computing and fuzzy clustering) work together to provide reliable segmentation results, characterized by a higher segmentation accuracy and robustness. The second is to reduce the computing complexity and speedup the segmentation process. By this work, we present two contributions as given below:

(1) A multiresolution image transformation based on 2-D Gabor filtering combined with a morphological gradient construction to generate a superpixel image with accurate boundaries. This proposition integrates a multiscale neighboring system to solve the problems of rotation, illumination, scale, and translation variance. This is very useful specially with images of high resolution.
(2) We introduce a Principal Component Analysis (PCA) to reduce the number of extracted Gabor features. From obtained regions we compute a simple color histogram to reduce the number of different intensities (pixels) and achieve a fast clustering for color image segmentation.

In summary, image segmentation methods can be roughly classified into two categories: supervised and unsupervised. In this paper, we mainly discuss a fuzzy unsupervised framework. No features learning is involved. This task remains one of the most challenging research topics because there is no unified approach to achieve fast, robust, and accurate segmentation.

In this work, a detailed study of existing color image segmentation approaches was carried out to investigate the most common stages in segmentation’s techniques. In Section 2, we discuss the motivation for using the different implemented techniques. Furthermore, we thoroughly described each phase and introduced ideas for improvements. Next, we describe the development of the proposed method. Section 4 presents an evaluation study of the proposed improvement using a set of synthetic and real color images from the well-known dataset (BSD 500 and MSRC). As a validation stage, the developed method is applied on fire forest images and compared to the standard and recent methods.

## 2. Motivation

### 2.1. Motivation for Using Superpixels with Gabor Filtering

In color image segmentation, non-texture areas are relatively uniform, and it is easy to obtain the accurate boundaries. Color and spatial information are sufficient for the clustering task. In texture areas, the boundaries are the combination of micro and macro adjacent regions. Here, texture edges cannot be incorporated only by characteristics of single pixels: intensities and spatial coordinates. Hence, to obtain these boundaries requires a combination of multi scale characteristics. Many researchers have verified that multi-resolution features are able to get the main outline for various texture regions of the image [12,13]. In the last decade, the Gabor filters, firstly proposed by Dennis Gabor in 1946 in 1-D and extended, in 1985, to 2-D by Daugman, have received much attention. Their wide usage in multiple fields can be taken as proof of their success: image analysis, compression and restoration, object tracking and movement estimation, face recognition, smoke detection, texture retrieval, contour extraction, or image segmentation [14,15,16,17].

### 2.2. Motivation for Using Color Images Histograms

**Figure 1:**
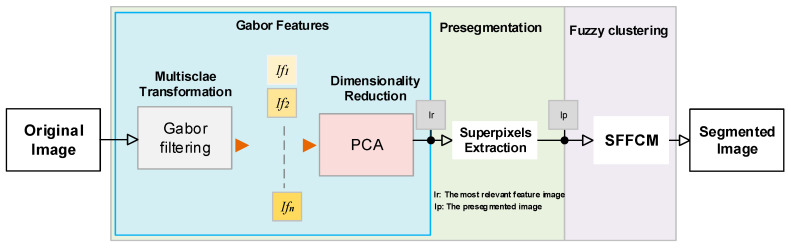
Overview of the proposed segmentation framework. Ifi is the $i^{th}$ image of Gabor features.

For C-means oriented algorithms, the clustering task has to compute the distance between each pixel and centers of different clusters. This task leads to a high computational complexity especially with images of higher resolution. Moreover, it is difficult to extend this idea of FCM for color image segmentation. This is due to the number of different colors which is usually close to the number of pixels in a color image. Compared to a grayscale image, the c-means clustering algorithms require a longer execution time to segment its corresponding color image. Because the histogram level is far less than the whole image pixels, the use of histogram-based features reduces the computational complexity of the clustering procedure. In [9], an enhanced FCM method for grayscale images was proposed. It is called the Spatial Fast Fuzzy C-means clustering algorithm (SFFCM). Authors demonstrate that it is faster to implement FCM on histogram-gray-levels than on pixel’s intensities. This novel extension of fuzzy clustering algorithm is used in our segmentation pipeline (see Figure 1).

### 2.3. Fire Forest Image Application

Recently, wildfires devasted millions of hectares over the world. The lack of information about the current state and the dynamic evolution of fire plays a central role in the accidents. Nowadays the demand increases for remote monitoring of this natural disaster [2,18,19,20]. For that, artificial visual control is a new area that has gained interest. In literature, many techniques have been developed mainly for wildfire image processing [4,8,21,22]. In real applications, for smoke and fire, there is a different useful information: area, location, direction, etc. Because the forest environment suffers from many perception field drawbacks (uncontrollable and sudden changes in environmental conditions, calibration problems, non-rigid fire-model, etc.), this study involves many advanced computer vision techniques in 2D [13] and extends them to the 3D domain [23]. Our project is divided into different research interests: image segmentation, semantic fire and smoke detection, and flame direction estimation. In this work we developed a color image segmentation technique as a part of mentioned tasks. The goal of the proposed method is to improve the segmentation performance of wildfire noisy images and to reduce the clustering computational complexity.

## 3. Methodology

The developed method is based on two principal tasks:

- The Pre-segmentation, also called the Superpixels Extraction,
- The Clustering of firstly extracted superpixels

The framework of our proposed algorithm is shown in Figure 1.

### 3.1. Superpixels Based on Gabor Filtering and Morphological Operations

#### 3.1.1. Superpixels Extraction: An Overview

Superpixels extraction, called also pre-segmentation, is the subdivision of the input image into a number of regions. Each region is a collection of pixels with homogenous characteristics. This procedure is always used for image classification and labeling. Compared to neighboring window-based methods, it is able to provide more representative local spatial information [9].

As given by [24], superpixel algorithms are classified into two principal categories:

*Graph-based methods:* each pixel is considered as a node in a graph. Similarities between neighboring pixels are defined as edge weights. Superpixels extraction minimizes a cost function defined over the graph. This category includes a large variety of developed methods: Normalized Cuts (NC), Homogeneous Superpixels (HS), Superpixels via Pseudo-Boolean Optimization (PB), and Entropy Rate Superpixels (ERS) [25,26].

*Clustering-based methods:* all image pixels are iteratively grouped until satisfying some convergence criteria. As given by [27], the most popular techniques are Simple Linear Iterative Clustering named (SLIC), Watersheds Transform (WT), Quick Shift (QS), and Turbo Pixel (TP). More details and evaluation of 15 superpixel algorithms are given in [24]. All mentioned approaches are usually considered as over-segmentation algorithms to improve the final segmentation. Referring to [9,27], in our work, we use the implementation of WT for the superpixels extraction. In the last part of experiments (Section 5.2), the SLIC is also implemented.

#### 3.1.2. Gabor Filters and Their Characteristics

Image filtering based on Gabor filters is a procedure widely used for the extraction of spatially localized spectral features. The frequency and orientation representation of Gabor filters are similar to human visual system, and they have been found vital features that can be used for image segmentation [16,28]. In our project, the processed images of fire forest combine many complexities due to the higher intensity’s variation and the texture geometrical diversity. To cope with complex image regions, we use a bank of filters as a multi-scale features extractor.

**Figure 2:**
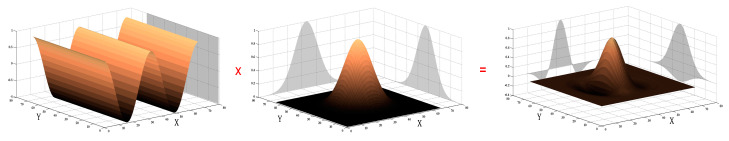
Spatial localization in 2D sinusoid (**left row**), Gaussian function (**middle row**), and corresponding 2D Gabor filter (**right row**).

The Gabor filter is obtained by a Gaussian kernel function modulated by multiplying a sinusoidal plane wave. As shown in Figure 2, combining a 2D sinusoid with a Gaussian function results in a 2D Gabor filter.

Gabor features are extracted by the convolution of the original image I(x,y) and the impulse response of the 2-D Gabor filter g(x,y):

(1) G(x,y)=I(x,y)⊗g(x,y)

*x* and *y* are the spatial coordinates of the plane.

The Gabor kernel generating g(x,y) is defined as follows:

As we have shown in [29], in the spatial domain, the 2-D Gabor function is formulated by:

(2) $$g_{\lambda ,\theta ,\varphi }(x,y)=e^{-\frac{{x^{\prime }}^{2}+\gamma^{2}{y^{\prime }}^{2}}{2\sigma^{2}}}\cos((2\pi \;x^{\prime }/\lambda )+\varphi ))$$

where

$$\begin{array}{l} x^{\prime }=x\cos\theta +y\sin\theta \\ y^{\prime }=-x\sin\theta +y\cos\theta \end{array}$$

**Figure 3:**
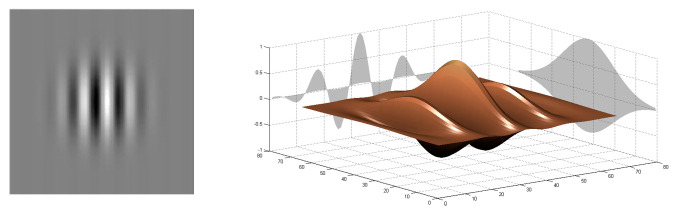
Example of the receptive field of the 2D-Gabor filter.

σ is the standard deviation of the Gaussian factor that determines the size of the receptive field. The parameter λ is the wavelength and F=1/λ the spatial frequency of the cosine factor. They are, respectively, called the preferred wavelength and preferred spatial frequency of the Gabor function. The ratio σ/λ determines the spatial frequency bandwidth and the number of parallel excitatory and inhibitory stripe zones that can be observed in the receptive field (see Figure 3).

γ is a constant, called the spatial aspect ratio, that determines the ellipticity of the receptive field.

θ represents the preferred orientation of the normal of the parallel stripes of a Gabor function, φ is the phase offset which defines the symmetry of Gabor filter.

As an example, with a different range of frequencies $f_{k}=\frac{\sqrt{2}}{k}\;\left(k=\{1,2,3\}\right)$ and orientations $\theta_{l}=l\cdot \left(\frac{\pi }{8}\right)\left(l=\{0,1,\ldots ,7\}\right)$, the convolution generates a Gabor feature matrix given by:

(3) $$G_{\left(f_{l},\theta_{k}\right)}=\left[\begin{array}{ccc} r(x_{0},y_{0}) & \cdots & r(x_{N},y_{0})\\ \vdots & \ddots & \vdots \\ r(x_{0},y_{M}) & \cdots & r(x_{M},y_{N}) \end{array}\right]$$

The set of 3 spatial frequencies and 8 equidistant orientations is applied. Each Gabor kernel size is proportional to the wavelength value. The replication padding is used to reduce boundary artifacts. For each specific pair of frequency and orientation $\left(f_{k},\theta_{l}\right)$, the feature image size is (M×N).

**Figure 4:**
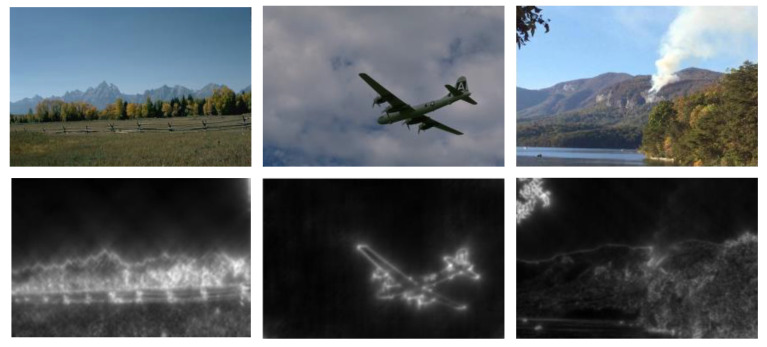
Example of object boundaries extraction using Gabor filters of $\left(f_{1},\;\theta_{3}\right)$. First row: original images. Second row: image of boundaries.

In our work, only the magnitude $r\left(x_{i},\;y_{j}\right)$ is considered. $r\left(x_{i},\;y_{j}\right)$ gives the intensity variations near the object boundaries (see Figure 4).

The Gabor features are processed with L2 normalization technique. The L2 norm is performed by:

(4) $$g\left(x,y\right)=\frac{\left\|G\left(x,y\right)\right\|}{max\left\{\left\|G\left(x,y\right)\right\|\right\}}$$

g is the normalized Gabor feature image.

#### 3.1.3. Gabor Feature Reduction Based on PCA

**Figure 5:**
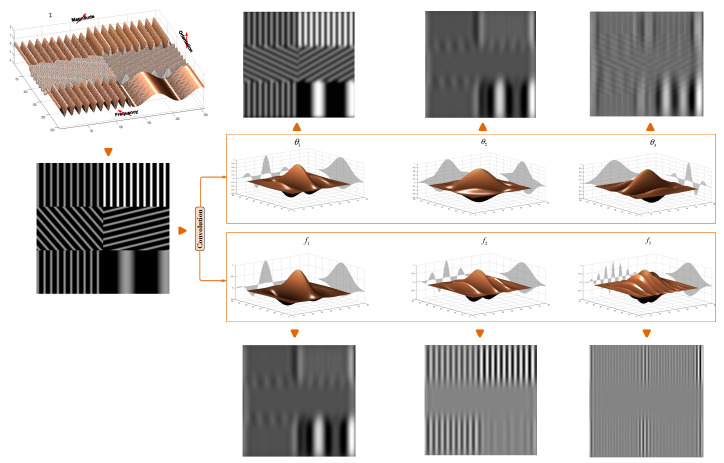
Convolution outputs of a synthetic image of sinusoids with various properties (orientations, frequencies and magnitudes) by Gabor filters of different frequencies and orientations $\left(f_{k},\theta_{l}\right)$.

High dimension data are extremely complex to process due to the inconsistences in the features which increase the computation time [30,31]. In our work, we only focus on the variation of frequency and orientation parameters of Gabor filters. In Figure 5, we present a convolution results of a synthetic image of sinusoids of different orientations, frequencies and magnitudes by Gabor filters of different orientations and frequencies.

Both the mentioned parameters (frequencies and orientations) generate a large feature dimension (K×L). As mentioned above, a set of K=3 different frequencies and L=8 orientations are considered producing 24 features for each position of the filter. This is not performant because of the redundancy of features due to correlation of the overlapping filters. Moreover, as illustrated in Figure 5, by comparing the convolution results we notice a higher sensibility of filter parametrization. Many researchers propose the use of a small bank of filters [30,31,32]. In this work, the problem of redundancy was addressed. Because its performance compared to other dimensionality reducer methods [32], we have used the PCA retaining only the most representative response of 24 outputs. It will be considered as the input of the superpixesls extraction stage (see Figure 1).

#### 3.1.4. Pre-segmentation Based on Gabor-WT

The WT produces a set of basins starting with a local minimal of a gradient image and searching lines between adjacent local minima that separate catchment watersheds. As given by [33], this is a relatively fast algorithm used for images with high resolution.

**Figure 6:**
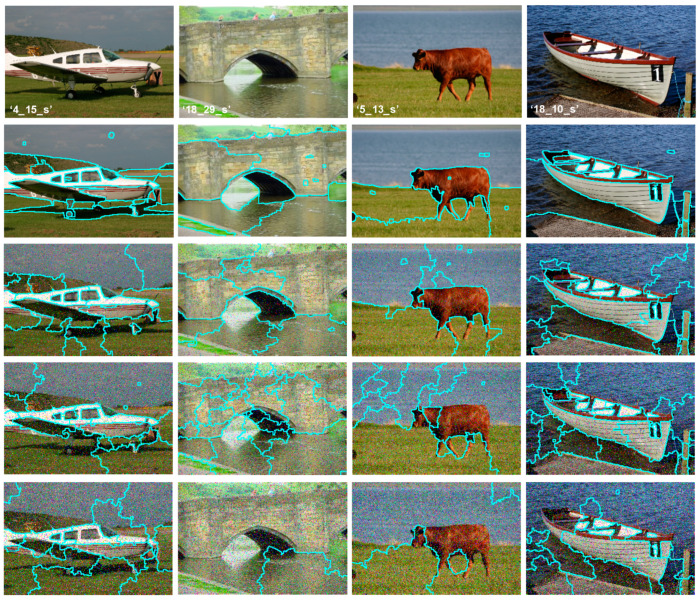
MMGR-WT robustness test. First row: original images from the MSRC-dataset. Second row: Superpixels extraction results with original images. 3rd, 4th, and 5th rows: the obtained results for corrupted images by (5%, 10%, 15%) salt and pepper noise.

For noisy image segmentation, to fulfill both regional consistency and boundary keeping simultaneously, become more and more difficult. As shown by Figure 6, the MMGR-WT, introduced in [9], causes an over-segmentation or under-segmentation because it is sensitive to added noise.

**Figure 7:**
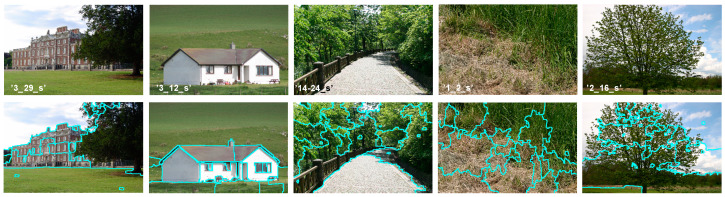
MMGR-WT superpixels extraction: test on uniform and textured regions. First row: original images from the MSRC_dataset. Second row: obtained results.

Moreover, these techniques greatly depend on the accurate extraction of region boundaries. The superpixels extraction performance of these methods deteriorates when the processed regions are textured or are of high varying intensities (see Figure 7).

As a summary of all the superpixels extraction given by Figure 6 and Figure 7, the MMGR-WT results exhibit major limits, namely the poor boundary keeping and superpixels consistency. This is clearly noticed with noisy images and ones of textured regions (grass, trees, sand, etc.). In our work, it should be noted that Fire Forest images suffer from all the general drawbacks (noise, higher textured regions, environmental conditions, etc.). In literature, many algorithms have been introduced to avoid such issues. Major methods tend to modify the gradient output of original image. In this paper, a 2-D Gabor filtering stage is used for the enhancement of the boundaries of regions for better superpixels extraction.

### 3.2. Fuzzy Superpixels Clustering

#### 3.2.1. Overview

A clustering divides data objects into homogeneous groups and performs a high similarity within a cluster (called compactness). Data partitioning is made according to a membership degree, in the range (0,1), which is proportional to the distance between the data and each cluster center. The partitioning result depends on the final centroid location [29]. The fuzzy oriented methods are based on the mentioned aspects and have been successfully used. For many an application, the traditional FCM clustering algorithm, firstly introduced by Bezdek, has depicted a higher performance. It is widely used for image segmentation. As an unsupervised clustering method, FCM does not need any prior knowledge about the image.

Let $X=\left\{x_{1},\;x_{2},\;\ldots ,\;x_{n}\right\}$ be a color image and nc be the number of clusters. Each $i^{th}$ image pixel belongs to the $j^{th}$ cluster with a fuzzy membership degree denoted by $u_{ij}$ according to its distance from the cluster center $v_{j}$. FCM can yield a good segmentation result by minimizing the following objective function:

(5) $$J_{FCM}={\sum_{i=}^{n}\sum_{j=}^{nc}\mu_{ij}^{m}\left\|x_{i}-v_{j}\right\|}^{2}$$

where $u_{ij}$ and $v_{j}$ are given as follows:

(6) $$u_{ij}={\left({\sum_{k=1}^{nc}\left(\frac{\left\|x_{i}-v_{j}\right\|}{\left\|x_{i}-v_{j}\right\|}\right)}^{\frac{2}{m-1}}\right)}^{-1}$$

(7) $$v_{j}=\frac{\sum_{j=1}^{n}u_{ij}^{m}x_{j}}{\sum_{j=1}^{n}u_{ij}^{m}}$$

and m is the degree of fuzziness.

The FCM algorithm is summarized in Algorithm 1.


**Algorithm 1.** Traditional FCM algorithm.
1:**Input:** X of *n* data to be clustered, the number of clusters *nc*, the convergence test ε>0 (or the max number of iteration), randomly cluster centers $v^{\left(t=0\right)}$, the fuzzifier m>12:**Output:** clustered data (pixel groups map)3:
**Begin**
4:  **Step 1.** Compute the membership matrix U by using Equation (6)5:  **Step 2.** Update the cluster centers $v^{\left(t+1\right)}$ with Equation (7)6:  **Step 3.** Test if $\left\|v^{\left(t+1\right)}-v^{\left(t\right)}\right\|<\varepsilon$, execute **step 4**; otherwise, t=t+1 and go to **step 1**7:  **Step 4.** Output the pixels group map8:
**End**




In literature, a large variety of modified versions of the FCM clustering algorithm have been proposed. In 2003, Zhang developed a new extension called KFCM by introducing the “Kernel method”. Later, in 2012, Zanaty et al. included a spatial information in the objective function of KFCM [34]. From 2005 to 2013 Pal et al. developed a possibilistic fuzzy clustering method called PFCM [35]. Another modification of FCM was proposed in 2015 by Zheng et al. [36] named the Generalized and Hierarchical FCM (GFCM), (HFCM). In 2017, in order to remove the information redundancy, Gu et al. proposed a novel version of FCM called SL-FCM [37]. Most of the mentioned methods are still time-consuming and unable to provide the desired segmentation accuracy. As mentioned above, Lei et al. developed the SFFCM algorithm. The modification is also based on the integration of the spatial information [9].

#### 3.2.2. The Proposed Clustering Method

**Figure 8:**
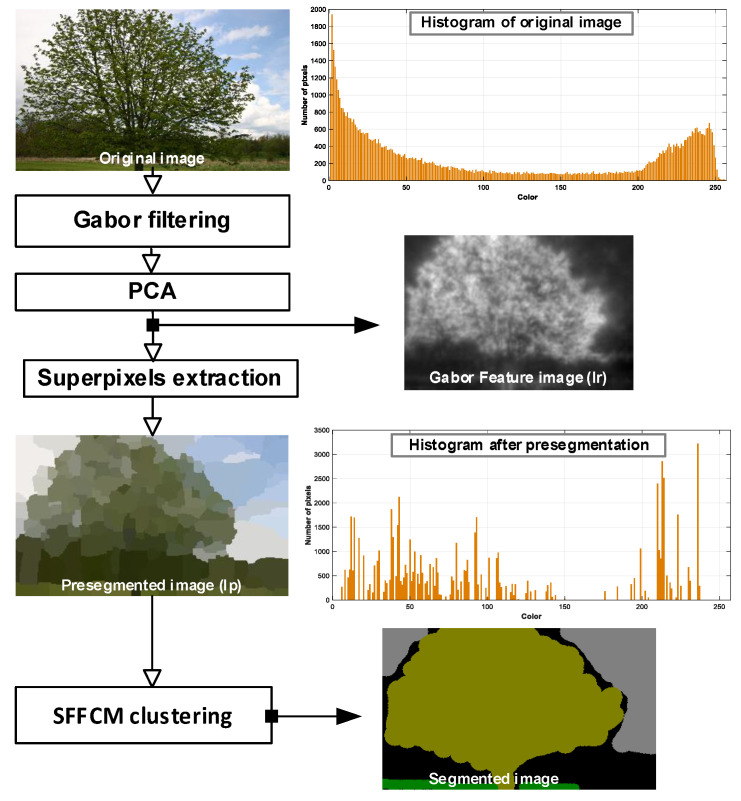
Example of the “End to End” segmentation pipeline with our proposed method.

Further to the time-consuming, FCM describes an image in terms of fuzzy classes. It only depends on global features. As given by Figure 8, we developed a Gabor-PCA superpixels-based method to extract the most representative local spatial information. By this, the input data to be clustered include only the subsegment levels. In our work, the proposed segmentation method has three goals. The first is to reach a higher robustness to added noise with the multiscale processing based on Gabor Filters. The second goal is the improvement of the segmentation accuracy by incorporating local features. The third, is reducing the computational complexity and time consuming by minimizing the size of data to be clustered.

In this paper, the SFFCM algorithm, firstly proposed by [9], is adopted. Adding the spatial information, the problem of fuzzily partitioning into nc clusters becomes formulated as the minimization of the objective function given by:

(8) $$J_{m}=\sum_{i=1}^{ns}\sum_{j=1}^{nc}S_{i}u_{ij}^{m}{\left\|Med_{i}-v_{j}\right\|}^{2}$$

With $Med_{i}$ the mean value of color pixels within the corresponding region $R_{i}$ of $i^{th}$ superpixel image given by:

(9) $$Med_{i}=\frac{1}{S_{i}}\sum_{p\in Ri}x_{p}$$

where:

ns is the number of superpixels, 1≤i≤ns the color level, and $S_{i}$ the number of pixels with color $x_{p}$ in $R_{i}$. The new objective function incorporates the histogram information by the level’s frequencies given by $S_{i}$. Thereby, each color pixel in original image is replaced by the mean value $Med_{i}$ of the region for which was assigned. The “*Med*-*image*” is called the pre-segmented image (see Figure 8).

New SFFCM objective function generates two novel formulation memberships ($u_{ij}$) and centroid functions ($v_{j}$) as follows:

(10) $$u_{ij}=\frac{{\left\|Med_{i}\;-\;v_{j}\right\|}^{\frac{-2}{m-1}}}{\sum_{k=1}^{nc}{\left\|Med_{i}\;-\;v_{k}\right\|}^{\frac{-2}{m-1}}}$$

(11) $$v_{j}=\frac{\sum_{i=1}^{ns}u_{ij}^{m}\sum_{p\in R_{i}}x_{p}}{\sum_{i=1}^{ns}S_{i}u_{ij}^{m}}$$

In Algorithm 2, we show the pseudo-code of the Spatial Fast Fuzzy C-means clustering method (SFFCM).


**Algorithm 2.** SFFCM Algorithm.
1:**Input:**$S=\{S_{1},\;\ldots ,\;S_{ns}\}$ number of pixels with color corresponding to the presegmented regions $R=\{R_{1},\;\ldots ,\;R_{ns}\}$, $Med=\{Med_{1},\;\ldots ,\;Med_{ns}\}$ the mean values of superpixels levels (equation …), the number of clusters nc, the convergence test ε>0 (or the max number of iteration), randomly cluster centers $v^{\left(t=0\right)}$, the fuzzifier m>12:**Output:** clustered data (pixel groups map)3:
**Begin**
4:  **Step 1.** Compute the membership matrix U by using Equation (10)5:  **Step 2.** Update the cluster centers $v^{\left(t+1\right)}$ with Equation (11)6:  **Step 3.** Test if $\left\|v^{\left(t+1\right)}-v^{\left(t\right)}\right\|<\varepsilon$, execute **step 4**; otherwise, t=t+1 and go to **step 1**7:  **Step 4.** Output the pixels group map8:
**End**




### 3.3. Evaluation Criteria

In the last decade, several metrics have been applied to evaluate the segmentation methods [38]. The major ones focus on segmentation accuracy, superpixels compactness, regularity, coherence, and efficiency. In [24], Wang et al. divided the set of metrics into three groups: segmentation quality evaluation, superpixels quality, and the efficiency measure based on runtime. In this work, two metrics categories are considered.

a. Segmentation accuracy

To test the clustering performance, we use two metrics given in [9]. The first measures the Equality Degree (ED) between Clustered Pixels (CP) and Ground truth Prediction (GP). The second measures the Segmentation Accuracy (SA) based on the sum of correctly classified pixels. Both metrics are, respectively, given by:

(12) $$ED=\sum_{k=1}^{nc}\frac{CP_{k}\cap GP_{k}}{CP_{k}\cup GP_{k}}$$

(13) $$SA=\sum_{k=1}^{nc}\frac{CP_{k}\cap GP_{k}}{\sum_{j=1}^{c}GP_{j}}$$

where, $CP_{k}$ is the set of pixels assigned to $k^{th}$ cluster and $GP_{k}$ the set of pixels belonging to the same class k of Ground Truth (GT)**.** nc denotes the number of clusters.

$CP_{k}\cap GP_{k}$: the comprised of the labeled pixels *AND* the ground truth of the $k^{th}$ cluster.

$CP_{k}\cup GP_{k}$: the comprised of all pixels found in either the prediction *OR* the ground truth of the $k^{th}$ cluster.

b. Sensitivity and Specificity

These measures are based on region overlapping. Here, two aspects are considered: the matching direction and the corresponding criteria. For the sensitivity measure, the matching direction is defined as a ground truth to segmentation result directional correspondence and vice versa for the recall measure. Sensitivity (SEN) and Specificity (SPE) are formulated as follows:

(14) $$SEN\left(CP,\;GP\right)=\frac{TP\left(CP,\;GP\right)}{TP\left(CP,\;GP\right)\;+\;FN\left(CP,\;GP\right)}$$

(15) $$SPE\left(CP,\;GP\right)=\frac{TN\left(CP,\;GP\right)}{TN\left(CP,\;GP\right)\;+\;FP\left(CP,\;GP\right)}$$

where:

TP(CP, GP)—*True Positives:* intersection between segmentation and ground truth
TN(CP, GP)—*True Negatives*: part of the image beyond the intersection mentioned above
FP(CP, GP)—*False Positives:* segmented parts not overlapping the ground truth
FN(CP, GP)—*False negatives*: missed parts of the ground truth

As given by the Equations (14) and (15), the quantitative evaluation based on Sensitivity (SEN) and Specificity (SPE) were performed between the (GT) and the clustering result. (SEN) was the percentage of Region of Interest (ROI) recognized by the segmentation method. (SPE) was the percentage of non-ROI recognized by the segmentation method.

Measures based on (SEN) and (SPE) are commonly used for the semantic segmentation. In our work, the mentioned metrics are applied to evaluate the clustering performance for supervised topics where the number of classes and region contents are known.

For real images, cluster frequencies are unbalanced. Mentioned metrics are not appropriate for evaluating because they are biased by the dominant classes. To avoid this, we have conducted the evaluation per-class. The obtained results are averaged over the total number of classes.

For multiclass, Sensitivity and Specificity are called Average True Positive Rate (Av_TPR) and Average True Negative Rate (Av_TNR) and given by:

(16) $$Av\_TPR=\frac{\sum_{k=1}^{nc}TP_{k}}{\sum_{k=1}^{nc}\left(TP_{k}+FN_{k}\right)}$$

(17) $$Av\_TNR=\frac{\sum_{k=1}^{nc}TN_{k}}{\sum_{k=1}^{nc}\left(TN_{k}+FP_{k}\right)}$$

## 4. Experimental Results

Experimental Setting

For all the experiments discussed below, the particular parameters for the compared methods are summarized in Table 1. Only Gabor filtering parametrization is detailed in [29].

In the first experiments, the tested images are synthetic with natural textures of Smoke, Fire, Sky, Sand, and Grass. For the second, we have tested on six real images from real scene of fire forest images. For the limited length of paper, in the last experiments, we only demonstrate the robustness and segmentation performance of our proposed method on a subset of twenty images from BSDS500 and MRSC datasets.

## 5. Application on Fire Forest Images

### 5.1. Results on Synthetic Images

**Figure 9:**
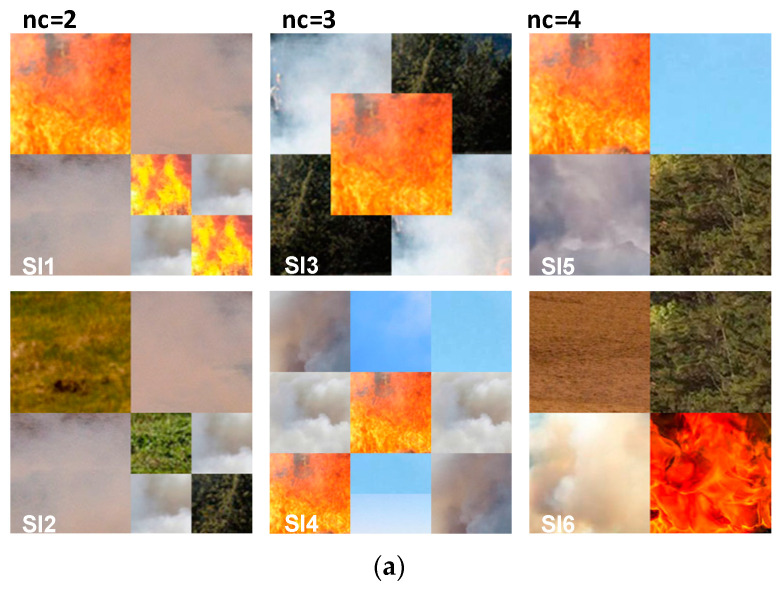

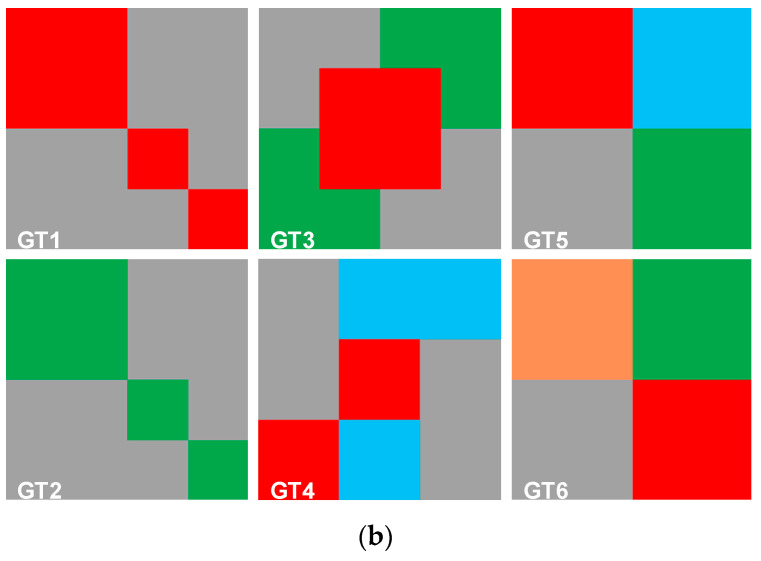
Synthetic test images of (256×256) pixels with manually created (GT). (**a**) Images with different regions of real contents. (**b**) The corresponding desired segmentation (GT).

At the first part of evaluation, we test the proposed method with the WT and the SFFCM algorithm on a set of six synthetic images shown by Figure 9a. For each class, (Fire, Smoke, Grass, Sand, Sky), the selected region is chosen from a random location in the original corresponding texture. All of the used synthetic images are with regions of regular boundaries. This is more suitable to manually generate the desired segmentation (see Figure 9b).

**Figure 10:**
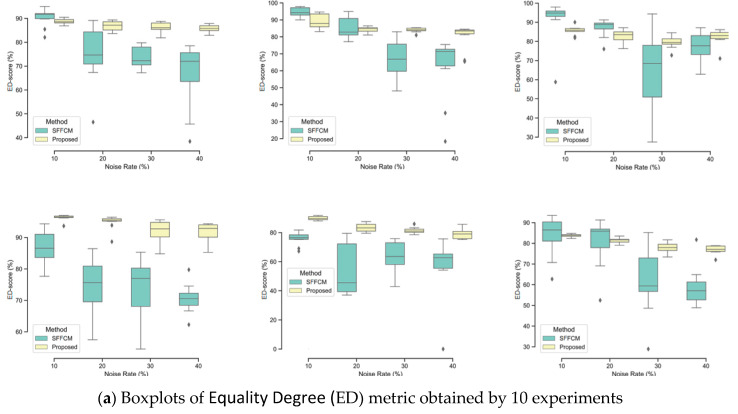

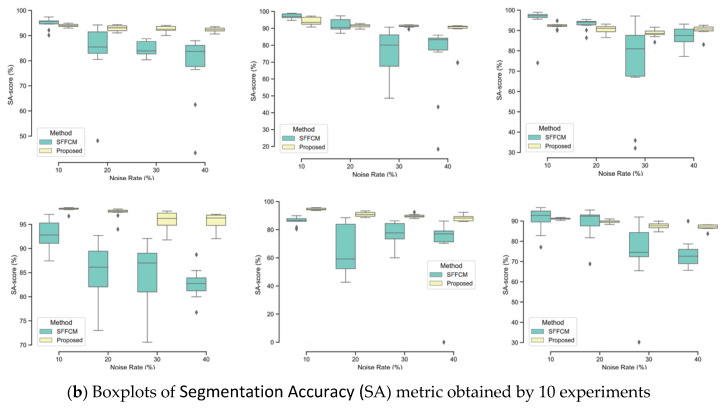
Comparison of SFFCM and our proposed method (*G-WT*) robustness. Application on the set of synthetic images SI1-SI6 (Figure 9) corrupted by the (10%, 20%, 30%, 40%) Gaussian noise.

**Figure 11:**
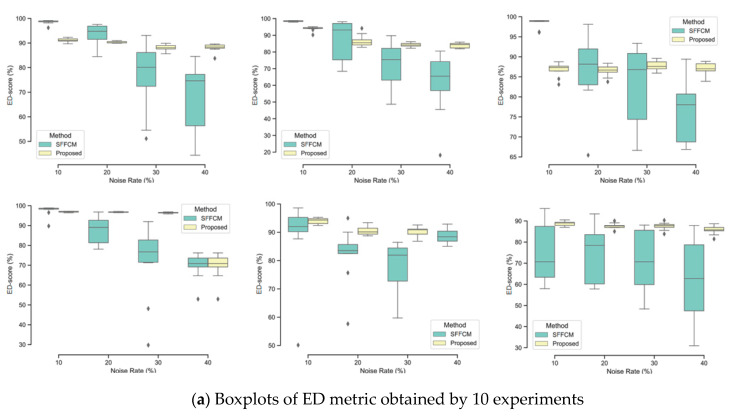

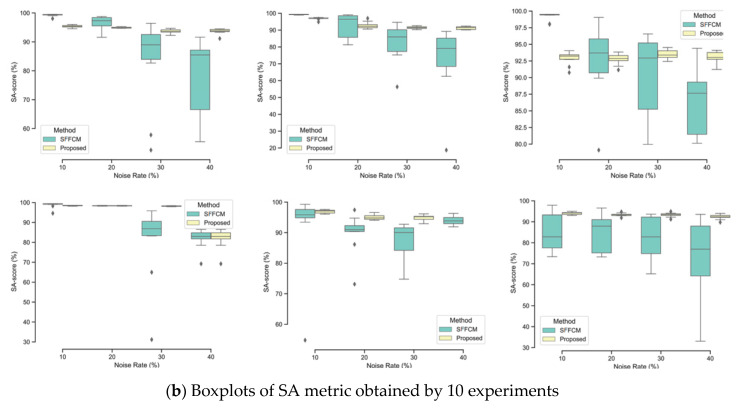
Comparison of SFFCM and our proposed method (*G-WT*) robustness. Application on the set of synthetic images SI1–SI6 (Figure 9 corrupted by the (10%, 20%, 30%, 40%) Salt and Pepper noise.

In this experiment, two types of noise are considered: Gaussian and Salt and Pepper. The robustness of each method is tested with four different densities of each kind of mentioned noises (10%, 20%, 30%, 40%). The quantitative segmentation results on the different blurred images achieved by using our developed method with WT and the SFFCM proposed by Lei 2019 [9]. Each experiment is repeated 10 times. All the obtained results are depicted in the boxplots in Figure 10 and Figure 11, reporting both ED and SA metrics values given by Equations (12) and (13). The graphs of boxplots arranged similarly as the map of images (SI1–SI6) given by Figure 9 (e.g., the top left boxplot corresponds to the results on image (SI1)).

In Figure 10 and Figure 11, the lower and the upper bounds of each boxplot represent the first and third quartiles of the distribution, respectively. The mean values of used metrics (ED, SA) are represented by a black solid line and outliers are displayed as black diamonds. We observe that there is a greater variability of the SFFCM results compared to our proposed method. Moreover, the boxplots pertaining to the proposed method results present the lowest statistical dispersion in terms of box height and number of outliers, thus implying a lower standard deviation compared to the SFFCM method. Therefore, the use of the novel method allows for considerably robust and accurate segmentation results.

### 5.2. Results on Real Images

**Figure 12:**
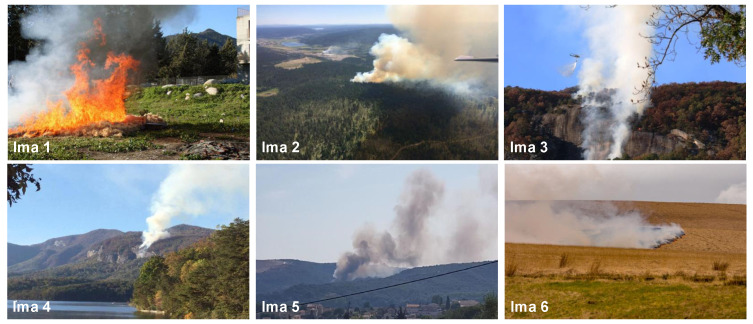
A set of real test fire forest images [21].

In addition to synthetic images, we shall evaluate the performance of our method on natural images. We apply the proposed method to the images from real fire forest sequences to examine the segmentation performance of our approach. The test images, given by Figure 12, are with different regions (fire, forest, smoke, cloud, grass, etc.). We shall assess the segmentation accuracy according to the visual inspection because no ground truths are available.

The difficulty of real image segmentation can be attributed to two reasons. The first is that image segmentation is a multiple solution problem. The number of clusters differs from a person to another. The second is that an image is always complex because of added noise and regions nonuniformity.

**Figure 13:**
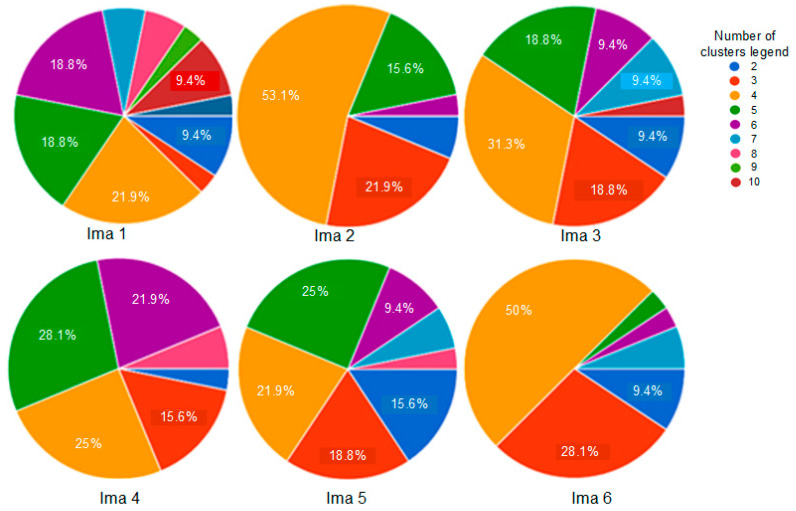
30 humans’ observations about the number of clusters of the real images given by Figure 12.

In this study, in order to address the first mentioned difficulty, we have shared the real test images with a group of 30 of our students in order to obtain their observations about the number of different observed clusters. The obtained statistics are summarized in Figure 13.

**Figure 14:**
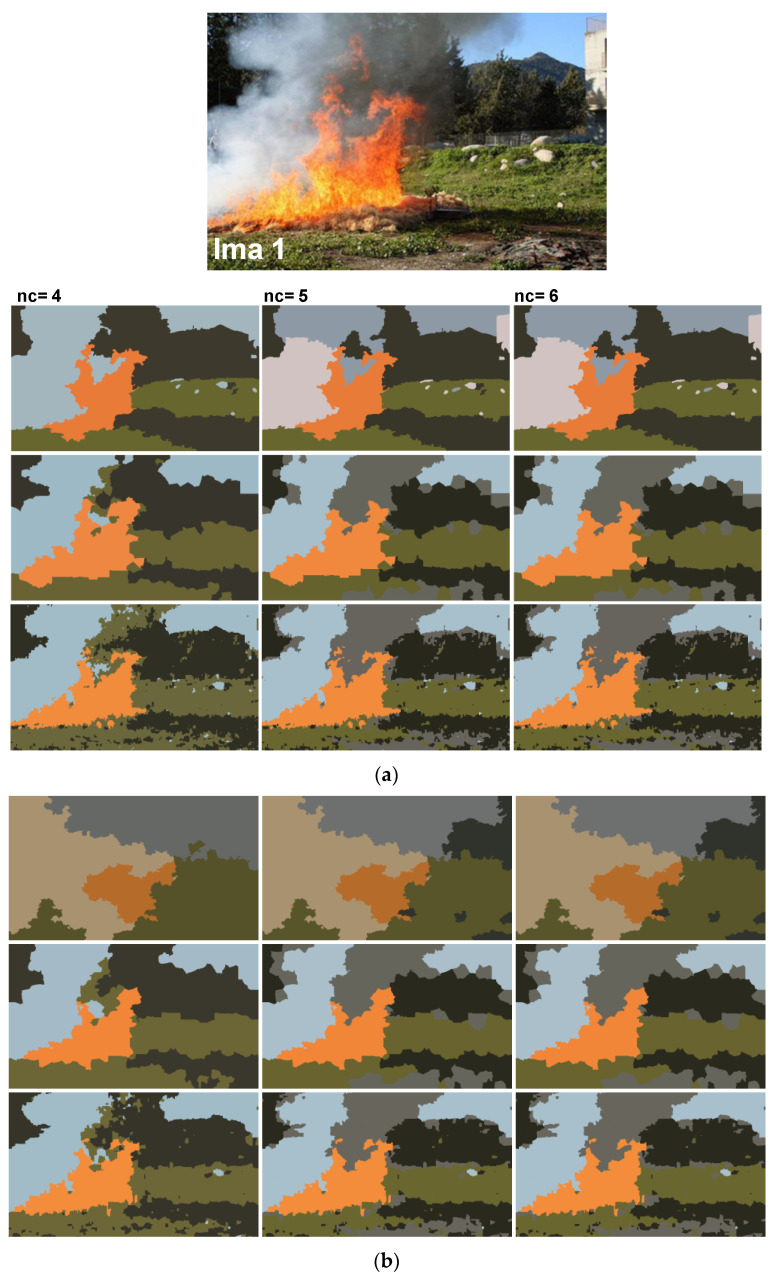
Comparison of segmentation results of original image Ima 1 (**a**) and corrupted by a 10% salt and pepper noise (**b**) obtained by: SFFCM (the first row), the proposed method based on Simple Linear Iterative Clustering (SLIC) (the second row), and the proposed method based on Watersheds Transform (WT) (the third row).

**Figure 15:**
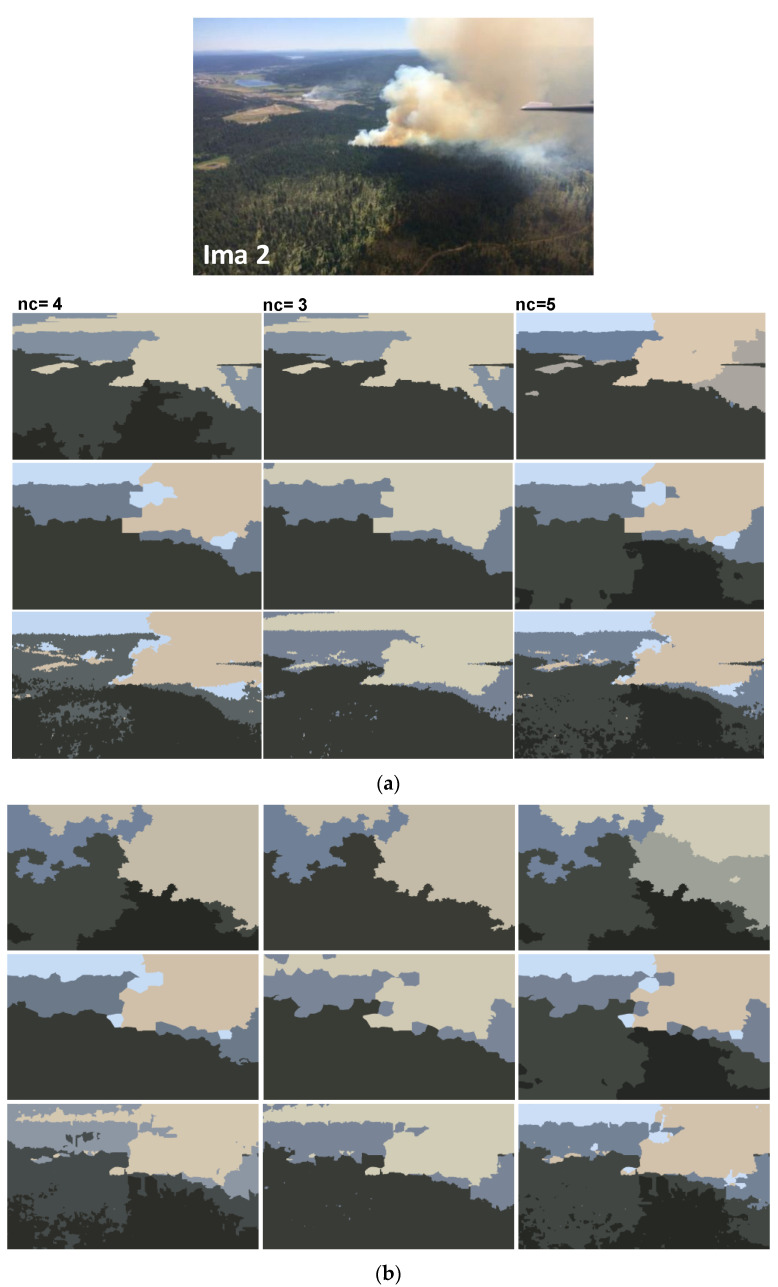
Comparison of segmentation results of original image Ima 2 (**a**) and corrupted by a 10% salt and pepper noise, (**b**) obtained by: SFFCM (the first row), the proposed method based on SLIC (the second row), and the proposed method based on WT (the third row).

**Figure 16:**
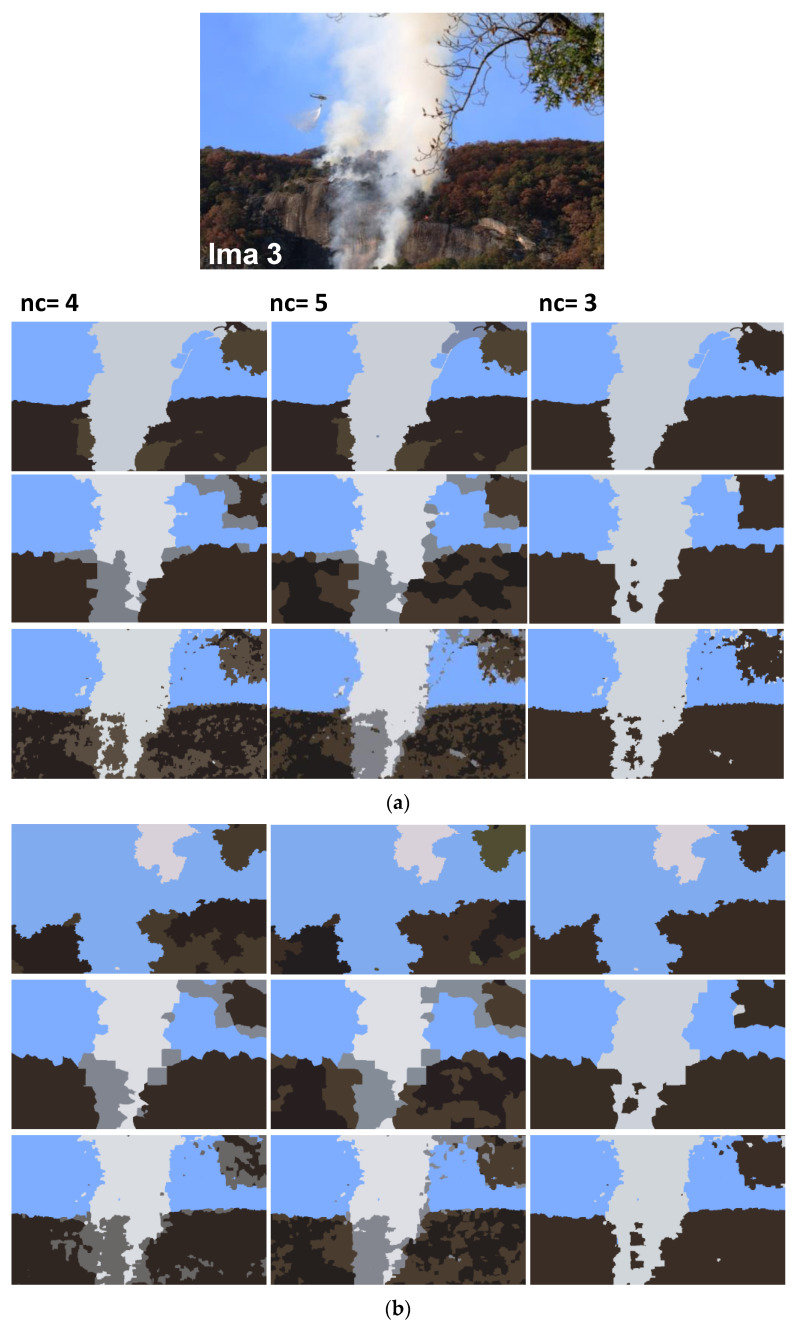
Comparison of segmentation results of original image Ima 3 (**a**) and corrupted by a 10% salt and pepper noise (**b**) obtained by: SFFCM (the first row), the proposed method based on SLIC (the second row), and the proposed method based on WT (the third row).

**Figure 17:**
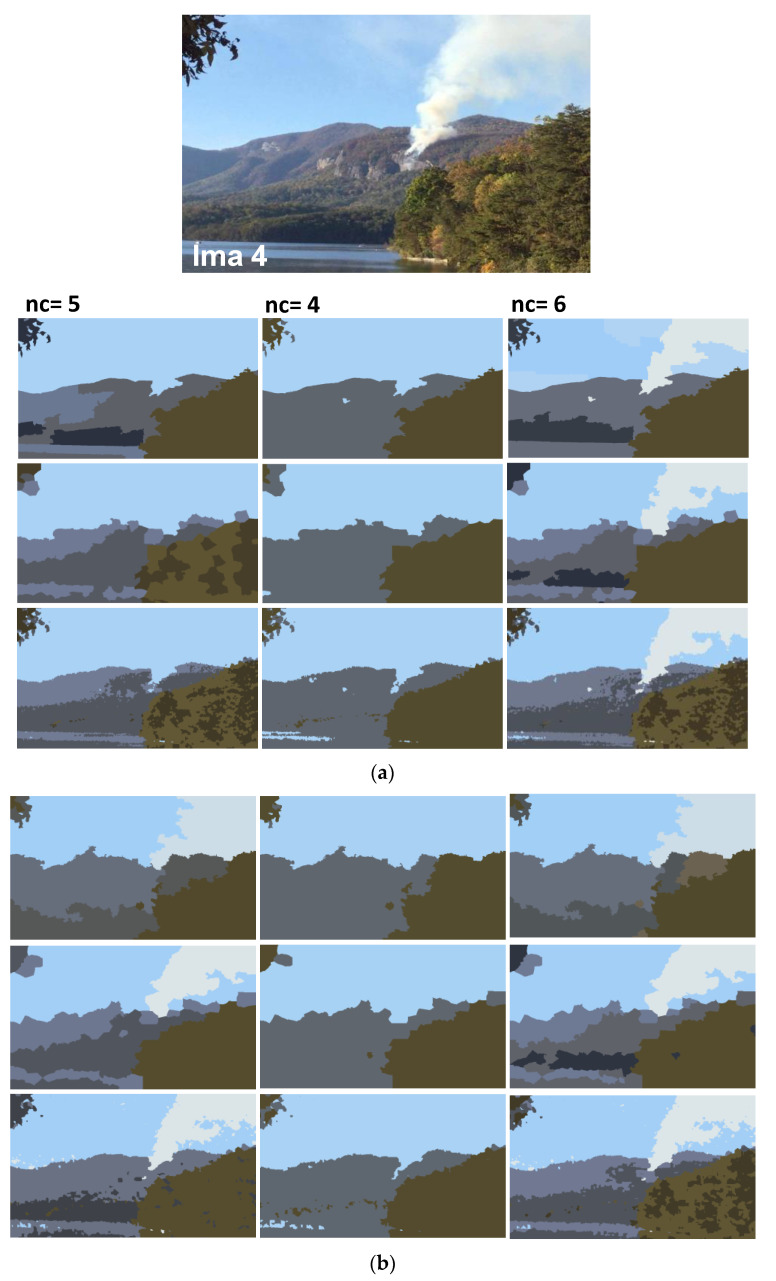
Comparison of segmentation results of original image Ima 4 (**a**) and corrupted by a 10% salt and pepper noise. (**b**) obtained by: SFFCM (the first row), the proposed method based on SLIC (the second row), and the proposed method based on WT (the third row).

**Figure 18:**
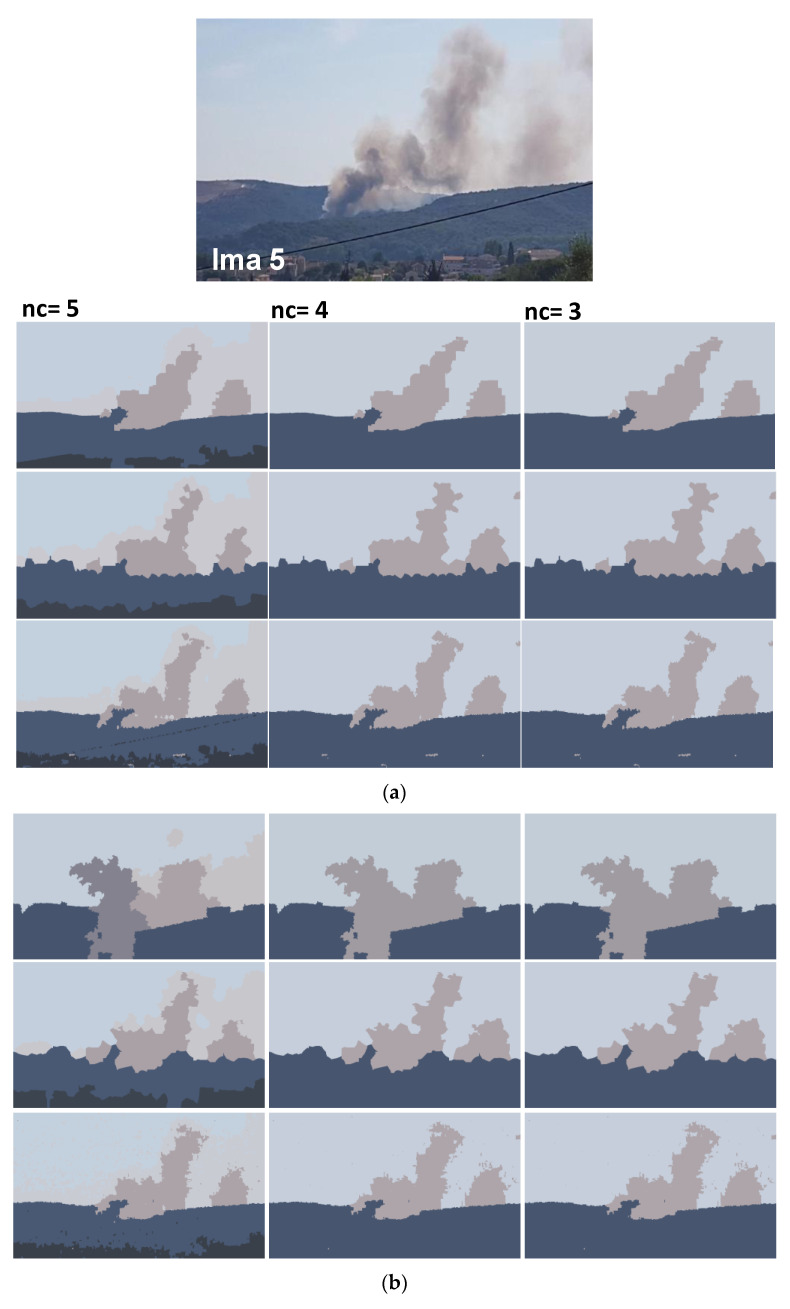
Comparison of segmentation results of original image Ima 5 (**a**) and corrupted by a 10% salt and pepper noise (**b**) obtained by: SFFCM (the first row), the proposed method based on SLIC (the second row), and the proposed method based on WT (the third row).

**Figure 19:**
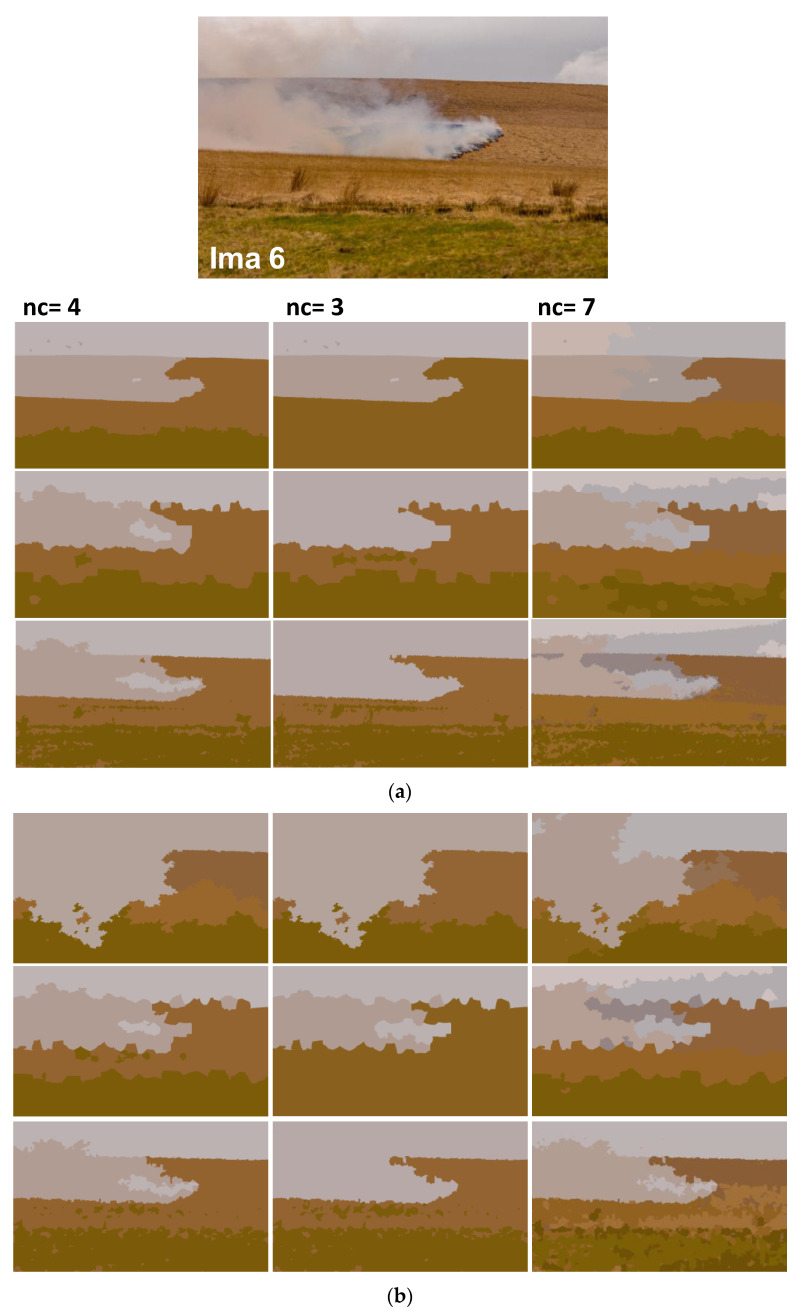
Comparison of segmentation results of original image Ima 6 (**a**) and corrupted by a 10% salt and pepper noise (**b**) obtained by: SFFCM (the first row), the proposed method based on SLIC (the second row), and the proposed method based on WT (the third row).

For each image, only the first three decisions with higher percentages were considered. i.e., as given by Figure 13, 53.1% of persons have considered that the image “Ima 2” is of 4 clusters, 21.9% have considered that the mentioned image is only of 3 clusters, and 15.6% observed that “Ima 2” is with 5 clusters. In our experiments, for mentioned image, we conduct the segmentation with 4, 3, and 5 clusters. All the obtained results are illustrated by Figure 14, Figure 15, Figure 16, Figure 17, Figure 18 and Figure 19.

Figure 14, Figure 15, Figure 16, Figure 17, Figure 18 and Figure 19 show the segmentation results of the real images depicted by Figure 12 and corrupted by the salt and pepper. In this experiment, we compare the SFFCM and our proposed method for two versions. The first by using the WT and for the second, we have introduced the SLIC pre-segmentation technique. By a visual inspection, for three compared methods, we notice that the region partition is satisfying. When the noise density is added, a lower performance is achieved. This is mainly due to the fact that high density of noise affects the texture structures, leading to the input image color degradation. Added noise affects the pre-segmentation performance and yields a lower classification performance. This clearly noticed with the SFFCM algorithm compared to the proposed method with WT and SLIC.

As given by Figure 14b, Figure 15b, Figure 16b, Figure 17b, Figure 18b and Figure 19b, for different corrupted images, the obtained results with the proposed method using WT depicts that the different regions separation is more accurate than using the SLIC. For instance, we can see that the “fire” in Figure 14b and the “smoke” in Figure 15b, Figure 16b and Figure 17b are accurately segmented.

In summary, the segmentation results obtained by the proposed method, using WT or SLIC, are still more satisfying. This is due to the higher robustness of the multiresolution transform based on Gabor filters and the integration of the PCA in the pre-segmentation stage.

### 5.3. Application on Other Natural Images

**Figure 20:**
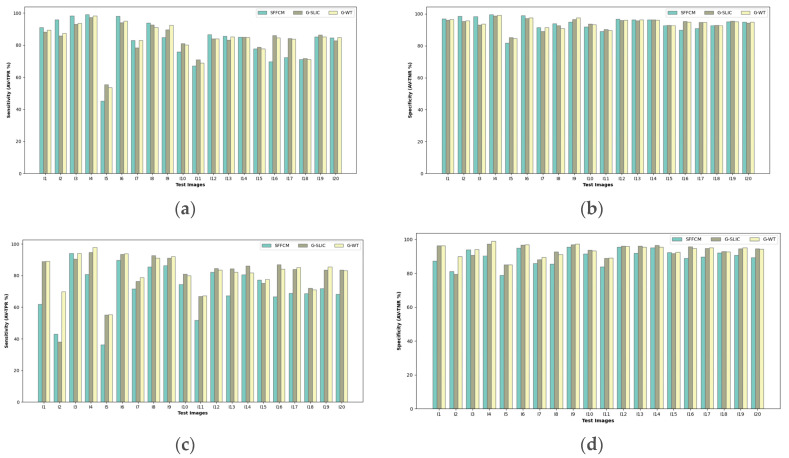
Comparison of SFFCM and our proposed method with WT and SLIC pre-segmentation techniques (G-WT, G-SLIC) based on averaged Sensitivity (**a**,**c**) and Specificity results (**b**,**d**) of 10 experiments. First row: test on natural images illustrated by Table 2. Second row: test on corrupted images with 10% Salt and Pepper noise.

To assess the performance of the proposed method, we further tested it on natural images from the BSDS and MSRC datasets (see Table 2). The both mentioned datasets are the most popular benchmarks and they are widely used by researchers for color image segmentation [27,39,40]. The results reported are averaged after 10 experiments and illustrated by Figure 20.

Referring to the barplots shown in Figure 20, a higher segmentation performance with the SFFCM is recorded. This superiority, of Sensitivity and Specificity, is clearly shown with original images (I1, I2, I3, I4, I6, I8, I12). For this image’s subset, it can be seen that different classes are with homogenous microtexture regions. By adding the salt and pepper noise, we can notice the degradation of the SFFCM segmentation accuracy compared to the proposed method. This robustness limitations of SFFCM was previously illustrated by the boxplots (see Figure 10 and Figure 11). Furthermore, it is clearly shown by Figure 20.

The selected images contain nonhomogeneous regions within the same class, and thus, grouping the superpixel regions in these cases would be a difficult task because these image blocks, which belong to the same group, are easily identified into two different groups. For instance, we can see the nonuniform texture patterns of “Trees” in images (I9, I10, I11, I13, I16, I17, I19). Nevertheless, the proposed method with WT (G-WT) reaches the higher degrees of true positive and true negative rates. This superiority is noted with original images and becomes greater for the case of blurred ones (see Figure 20). This is because our superpixel approach is based on Gabor filtering which is effective for macro texture characterization. This is was proved in our previous work [29].

The obtained results show that the segmentation with MMGR-WT proposed by Lei et al. gives the best Sensitivity and Specificity only for images with homogeneous regions. It is still with lower performance for images with textured regions of higher intensities variations (e.g., cloud, trees, grass). As a summary of all the obtained results, it is clearly noticed that the proposed is more performant for our application on fire forest images. Where, for the major cases, the different regions are with a large texture variety and higher nonhomogeneous regions (e.g., smoke, fire, trees, grass, etc.).

## 6. Conclusions and Future Works

Segmentation is an important topic in the image processing community. In this study, we presented an end to end framework for application in fire forest image segmentation. The proposed approach is divided into two principle stages: the pre-segmentation and the fuzzy clustering. Our main contributions are in the pre-segmentation stage. First, we have applied a multiscale transformation based on Gabor filtering to improve the superpixel extractions. Second, for the variety of outputs generated by the different pairs of frequencies and orientations (24 filters), we have introduced the PCA to fulfill the dimensionality reduction. The goal is to keep only the most relevant output to improve the regional consistency at the end of presegmentation stage. The clustering is processed by the fuzzy method recently proposed by Lei et al.

The comparison results discussed above show the efficiency of the novel approach. This is clearly shown with images of nonhomogeneous regions. The robustness of the proposed method is experimentally justified by all the above segmentation results on a set of blurred images with different kinds and intensities of noise.

It is worth noting that, generally, our proposed method gives promising image segmentation performance, but it suffers from some shortcomings. First, a few parameters in the algorithm need to be selected appropriately so as to achieve satisfactory results (e.g., Gabor filter frequencies and orientation). Second, the first stage of pre-segmentation (i.e., Gabor filtering and PCA features reduction) is computationally expensive compared to the SFFCM method. Thus, it would be a future work on a fast and effective method can be used with fire forest images. Moreover, the fire and smoke are identified based on the range of color intensities. To improve the automatic fire and smoke detection, a semantic segmentation will be performed by introducing the Deep Learning techniques.

## Figures and Tables

**Table 1:** Parameters of different applied methods.

Method	Pre-segmentation	Classification
SFFCM [9]	Min, Max radius: $\left(r_{1},\;r_{2}\right)=(1,\;10)$	m=2, $\varepsilon =10^{-3}$
Gabor-SLIC	Number of desired pixels: $s_{k}=500$ Weighting factor: $s_{m}=50$ Threshold for region merging: $s_{s}=1$	m=2, $\varepsilon =10^{-3}$
Gabor-WT	Structured Element SE: a disk Radius: r=5	m=2, $\varepsilon =10^{-3}$

**Table 2:** Natural Images from BSDS 500 and MSRC datasets.

Image	Name	Dataset	Images
I1	“55067”	BSDS500	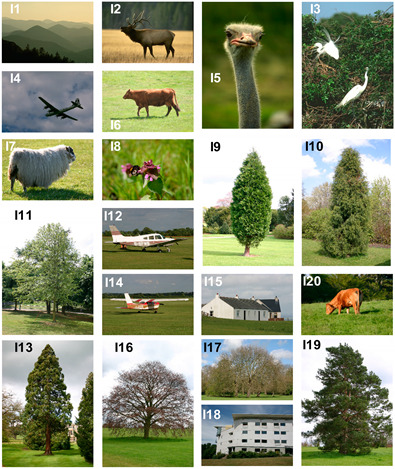
I2	“41004”		
I3	“311068”		
I4	“3096”		
I5	“66075”		
I6	“5_26_s”	MSRC	
I7	“9_10_s”		
I8	“10_1_s”		
I9	“2_21_s”		
I10	“2_22_s”		
I11	“2_27_s”		
I12	“4_13_s”		
I13	“2_8_s”		
I14	“4_26_s”		
I15	“3_20_s”		
I16	“2_17_s”		
I17	“2_3_s”		
I18	“3_24_s”		
I19	“2_20_s”		
I20	“5_22_s”		
